# Impact of time to first relapse on long-term outcome in adult retroperitoneal sarcoma patients after radical resection

**DOI:** 10.1007/s10147-022-02205-w

**Published:** 2022-06-28

**Authors:** Huajie Guan, Mengmeng Liu, Shaohui Cai, Biyi Ou, Yuanxiang Guan, Yao Liang

**Affiliations:** 1 State Key Laboratory of Oncology in South China, Collaborative Innovation Center for Cancer Medicine, Sun Yat-Sen University Cancer Center, 651 Dongfeng East Road, Guangzhou, 510060 China; 2 Department of Gastric Surgery, Sun Yat-Sen University Cancer Center, 651 Dongfeng East Road, Guangzhou, 510060 China; 3 Department of Melanoma and Sarcoma Medical Oncology, Sun Yat-Sen University Cancer Center, 651 Dongfeng East Road, Guangzhou, 510060 China

**Keywords:** Retroperitoneal sarcoma, Time to local recurrence, Survival, Prognostic factors

## Abstract

**Background:**

Local recurrence of primary retroperitoneal sarcoma (RPS) is one of the major causes of treatment failure and death. We attempted to assess the effects of time to local recurrence (TLR) on the survival after recurrence (SAR) and overall survival (OS) of RPS.

**Methods:**

Included in this study were 224 patients who underwent R0 resection for primary RPS at our institution between January 2000 and December 2020, 118 of whom had local recurrence. Based on the median TLR (19.8 months), patients were divided into two groups: early local recurrence (ELR < 20 months) and late local recurrence (LLR > 20 months). The Kaplan–Meier method was employed to calculate the local recurrence-free survival (LRFS), SAR and OS. Univariate and multivariate analyses were conducted to explore the prognostic value of TLR.

**Results:**

The median follow-up time was 60.5 months for the entire cohort and 58.5 months for the recurrence cohort. There were 60 (50.8%) patients in the ELR group and 58 (49.2%) in the LLR group. The ELR group exhibited a worse SAR (29.2 months vs. 73.4 months, *P* < 0.001), OS (41.8 months vs. 120.9 months, *P* < 0.001), and a lower 5-year OS rate (35.9% vs. 73.2%, *P* = 0.004) than the LLR group. Furthermore, multivariate analysis indicated that TLR was an independent prognostic indicator for SAR (*P* = 0.014) and OS (*P* < 0.001).

**Conclusions:**

In patients with RPS, ELR after R0 resection presents adverse effects on OS and SAR than those with LLR, and TLR could serve as a promising predictor for OS and SAR.

**Supplementary Information:**

The online version contains supplementary material available at 10.1007/s10147-022-02205-w.

## Introduction

Soft tissue sarcomas (STS) are a wide group of rare cancers of mesenchymal origin, characterized by high heterogeneity and various pathological subtypes [1]. Retroperitoneal sarcomas (RPS) account for 15% of all STS [2], of which the 5-year overall survival (OS) rate is between 52 and 66.6% [3–5]. Extensive resection is the main means of radical treatment for patients with RPS [6, 7]. However, due to the malignant behavior of RPS and the complexity of the anatomical structure of the site, the local recurrence rate of RPS within 5 years after the first operation is greater than 50% [3, 6]. Moreover, the 5-year OS rate was reduced by approximately 10% when there was local recurrence of tumors [8, 9]. Therefore, recurrence after surgical resection is the most common cause of treatment failure in patients with RPS.

In recent decades, there has been a wide range of literature suggesting that local recurrence can be a major factor in poor postoperative outcomes in patients with a variety of tumors [10–12]. Further studies found that the time to local recurrence (TLR) varied in different types of cancer, resulting in different rates of survival after recurrence (SAR). According to the duration of postoperative recurrence, TLR can be divided into early local recurrence (ELR) and late local recurrence (LLR). It was reported that there is a more optimal prognosis for primary breast sarcoma, renal cell carcinoma, and oral squamous cell carcinoma patients with LLR than those with ELR [13–15]. However, the prognostic value of TLR and the predictive factors of SAR in RPS have not been well demonstrated in the literature.

Therefore, we performed this retrospective study to investigate the potential factors that influence local recurrence and SAR in primary RPS patients who received complete resection, and identify the prognostic value of TLR for estimating survival.

## Patients and methods

### Study population

We retrieved clinicopathological data for 308 patients who underwent resection for primary RPS from January 2000 to December 2020 at the Sun Yat-sen University Cancer Center (SYSUCC, Guangzhou, China). Patients were excluded: (1) who had incomplete medical records; (2) who presented with metastasis at the initial diagnosis or were without R0 resection; (3) who were younger than 18 years of age; (4) who were lost to follow-up. The definition of R0 resection was microscopic absence of malignant cells at the resection margin according to the standardized classifications of the International Union Against Cancer (UICC) for surgery to classify the radicality of the surgical resections performed [16]. The final cohort consisted of 224 patients (Figure S1).

This study was approved by the institutional review board of SYSUCC (No. B2021-314-01), and the ethics committee decided that it was unnecessary to obtain patient informed consent. All patient data used were anonymously analyzed.

### Data collection

The clinical and pathological data for all included patients were retrospectively collected from the patients’ medical records at first diagnosis, such as gender, age, tumor size, and tumor stage. Multifocality was defined as more than one noncontiguous tumor through pathological confirmation, and the diameter of the multifocal tumors was measured by the long axial of the biggest specimen. The histological grade was determined based on the Fédération Française des Centres de Lutte Contre le Cancer (FNCLCC) grading system [17], and tumor stage was classified using the American Joint Committee on Cancer (AJCC) TNM staging system (8th version) [18].

The authenticity of this article was validated by uploading the key raw data to the Research Data Deposit public platform (www.researchdata.org.cn) with the approval RDD number of RDDA2021124250.

### Follow-up

All patients had been assessed with physical examination, computerized tomography (CT), or magnetic resonance imaging (MRI) every 3–6 months within the first 2 years after discharge and every 12 months thereafter. Continued follow-up was performed via out-patient clinic visits or regular telephone interviews conducted by the independent follow-up department of SYSUCC. The final follow-up time was considered the latest follow-up date of this study (September 1, 2021) or death.

Local recurrence was defined as the first relapse of the sarcoma at the site of a primary tumor, occurring more than 3 months after initial surgical resection, and detected by radiological examination, physical examination, or clinical symptoms. TLR was defined as the time from primary surgery to the first local recurrence. SAR was the duration between the day of the first local recurrence and the day of the last follow-up or the day of death. OS was defined as the interval between the date of the operation to the date of death from any cause or last follow-up.

### Statistical analysis

Categorical variables are expressed as a number (%), and continuous variables are summarized as median values (interquartile ranges, IQRs). Comparisons of variables between groups were performed using the Chi-square test, Fisher’s exact test for categorical data, or the Mann–Whitney *U*-test for continuous variables. Survival data was estimated by the Kaplan–Meier method, and the differences in survival were compared with the log-rank test. Independent risk factors associated with local recurrence, SAR, and OS were investigated using the Cox proportional hazard model. Hazard ratios (HRs) estimated from the Cox analysis were reported as relative risks with a corresponding 95% confidence interval (CI). A *p* value less than 0.05 was considered statistically significant with two-sided statistical tests. All the data analyses were performed using IBM SPSS statistics version 20.0 software (SPSS Inc., Chicago, IL, USA).

## Results

### Baseline clinicopathological characteristics

As shown in Table 1, there were 106 (47.3%) males and 118 (52.7%) females with a median age of 53 years (IQR 44.3–61.0 years). The median diameter of the tumors was 14 cm (IQR 8.1–20 cm). Dedifferentiated liposarcoma (DDLPS, *n* = 70, 31.3%), well-differentiated liposarcoma (WDLPS, *n* = 64, 28.6%), and leiomyosarcoma (LMS, *n* = 39, 17.4%) comprised of most of the histological subtypes of these cases, and detailed characteristics compared by different histology types are listed and compared in Table S1. There were 53 (23.7%), 101 (45.1%), and 70 (31.3%) patients with FNCLCC grade 1, 2, and 3, respectively. Furthermore, most patients presented with locally advanced tumors, which were specific in AJCC stage IIIA (*n* = 44, 19.6%) and IIIB (*n* = 109, 48.9%), and were found in more than half of the entire cohort. Multifocal tumors were present in 37 (16.5%) patients. Combined organ resection had been performed on 103 patients, 77 of which had one organ and 26 had more than one organ resected. Postoperative therapy as chemotherapy, radiotherapy, and combined chemoradiotherapy was administered to 38 (17.0%) patients, 6 (2.7%) patients, and 3 (1.3%) patients, respectively. In addition, 177 (79.0%) patients did not receive any postoperative therapy.

**Table 1:** Baseline characteristics of all patients (*n* = 224)

Characteristics	Cases	Percentage (%)
Smoking		
With	35	15.6
Without	189	84.4
Gender		
Male	106	47.3
Female	118	52.7
Age at operation (years)		
Median (interquartile range)	53 (44.3–61.0)	–
< 60	69	30.8
≥ 60	155	69.2
Body mass index (kg/m^2^)		
< 18.5	26	11.6
≥ 18.5 to < 24.0	137	61.2
≥ 24.0	61	27.2
Pathological types		
WDLPS	64	28.6
MLPS	20	8.9
DDLPS	70	31.3
LMS	39	17.4
MFH	11	4.9
Others^a^	20	5.9
Tumor size (cm)		
Median (interquartile range)	14 (8.1–20.0)	
< 15	117	55.2
≥ 15	107	47.8
T classification		
T1	21	9.4
T2	58	25.9
T3	52	23.2
T4	93	41.5
*N* classification		
N0	219	97.8
N1	5	2.2
FNCLCC grade		
G1	53	23.7
G2	101	45.1
G3	70	31.3
AJCC stage, 8th ed		
I	51	22.8
II	20	8.9
IIIA	44	19.6
IIIB	109	48.7
Number of resected organs		
0	121	54.0
1	77	34.3
≥ 2	26	11.6
Multifocality		
No	187	83.5
Yes	37	16.5
End-point		
Alive	157	70.1
Dead	67	29.9
Local recurrence		
No	106	47.3
Yes	118	52.7
ELR	60	50.8
LLR	58	49.2
Metastasis after operation		
No	198	88.4
Yes	26	11.6
Metastasis after recurrence		
No	105	89.0
Yes	13	11.0
Postoperative therapy		
None	177	79.0
Chemotherapy	38	17.0
Radiotherapy	6	2.7
Combined chemoradiotherapy	3	1.3
Therapy after recurrence		
None	11	9.3
Surgery alone	77	65.3
Chemotherapy alone	6	5.1
Targeted therapy alone	1	0.8
Combined chemoradiotherapy	2	1.7
Surgery + chemotherapy	9	7.6
Surgery + radiotherapy	3	2.5
Surgery + chemoradiotherapy	4	3.4
Surgery + targeted therapy	5	4.2

*WDLPS* well-differentiated liposarcoma, *MLPS* myxoid liposarcoma, *DDLPS* dedifferentiated liposarcoma, *LMS* leiomyosarcoma, *MFH* malignant fibrous histiocytoma, *FNCLCC* French National Federation of the Centers for the Fight Against Cancer, *AJCC* American Joint Committee on Cancer, *ELR* early local recurrence, *LLR* late local recurrence

^a^Including fibrosarcoma, synoviosarcoma, rhabdomyosarcoma, solitary fibrous tumor

### Local recurrence and influencing factors

Distant organ metastasis after surgery developed in 26 patients (11.6%), and 118 (52.7%) were diagnosed with local recurrence as their first event. The median RFS was 45 months, and the RFS rates at 3 and 5 years were 58.6% and 40.6%, respectively. The Kaplan–Meier curve analysis indicated that the 5-year OS rates of patients who developed local recurrence were significantly lower than those who did not develop local recurrence (58.7% vs 90.2%; *P* < 0.001; Figure S2).

**Fig. 1:**
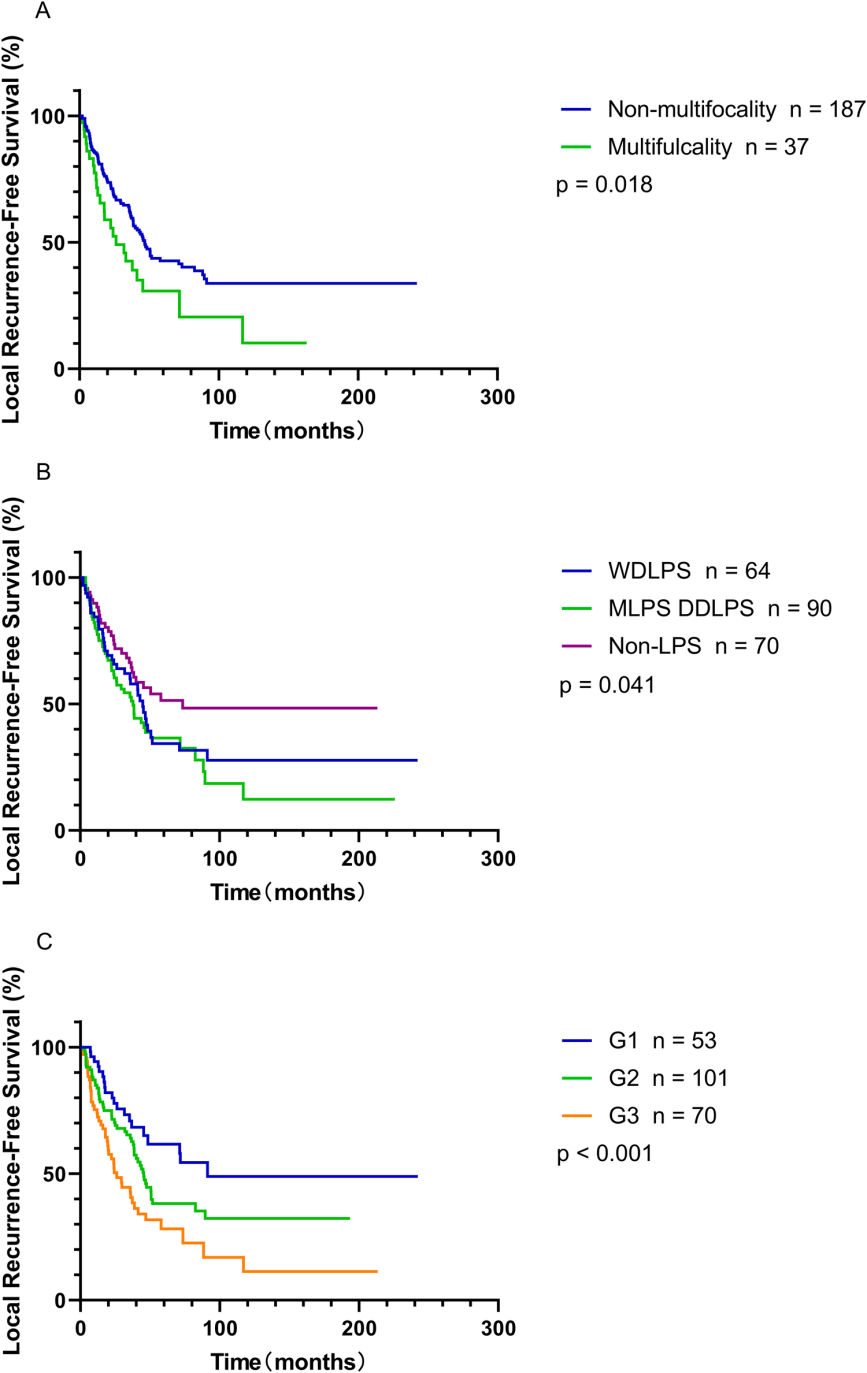
LRFS compared between different groups of patients using the Kaplan–Meier method and log-rank regression analysis. **A** The curves showed that the patients with multifocality exhibited significantly worse LRFS than those without multifocality (*P* = 0.02). **B** The curves showed that the patients with non-liposarcoma had significantly greater LRFS than those with WDLPS, and MLDPS/DDLPS (*P* = 0.04). **C** The curves showed that the patients with G1 tumors exhibited significantly higher RFS than those with G2 tumors, and the worst prognosis was for those with G3 tumors (*P* < 0.001)

Multivariate analysis showed that the multifocal status of the primary tumor (yes vs. no, HR = 1.62, 95% CI 1.02–2.57, *P* = 0.04), pathological subtypes (DDLPS/MLPS vs. non-liposarcoma, HR = 2.75, 95% CI 1.59–4.77; WDLPS vs. non-liposarcoma, HR = 1.60, 95% CI 1.00–2.55, *P* = 0.002), and histopathological grading (G2 vs. G1, HR = 2.50, 95% CI 1.40–4.47; G3 vs. G1, HR = 4.50, 95% CI 2.38–8.50; *P* < 0.001) were significantly associated with local recurrence. However, there were no differences in the incidence of local recurrence between the patients with or without postoperative treatments (*P* = 0.287) and tumor diameter with or without ≥ 15 cm (*P* = 0.131) (Table 2, Fig. 1).

**Table 2:** Univariate and multivariate analyses to determine independent predictors for LRFS of RPS

Variables	Local recurrence-free survival						
	Univariate				Multivariate		
	HR	95% CI	*P* value		HR	95% CI	*P* value
Gender (male vs. female)	1.263	0.880–1.814	0.205				
Age at operation (≥ 60 years vs. < 60 years)	1.370	0.924–2.033	0.118				
Maximal size of tumor (≥ 15 cm vs. < 15 cm)	1.055	0.735–1.515	0.772				
Multifocality (yes vs. no)	1.713	1.092–2.685	0.019		1.620	1.022–2.568	0.040
Number of resected organs (≥ 1 vs. 0)	0.880	0.612–1.265	0.490				
Histology subtypes			0.044				0.002
DDLPS/MLPS vs. Non-liposarcoma^a^	1.788	1.132–2.822			2.752	1.587–4.772	
WDLPS vs. non-liposarcoma^a^	1.513	0.930–2.462			1.598	1.002–2.548	
FNCLCC grade			0.001				< 0.001
G2 vs. G1	1.638	0.979–2.742			2.500	1.399–4.468	
G3 vs. G1	2.649	1.559–4.502			4.501	2.384–8.500	
AJCC stage			0.024				
II vs. I	1.547	0.741–3.230					
III vs. I	1.961	1.203–3.194					
Postoperative therapy (yes vs. no)	1.388	0.908–2.123	0.130				

*LRFS* local recurrence-free survival, *RPS* retroperitoneal sarcoma, *HR* hazard ratio, *95% CI* 95% confidence interval, *DDLPS* dedifferentiated liposarcoma, *MLPS* myxoid liposarcoma, *WDLPS* well-differentiated liposarcoma, *FNCLCC* French National Federation of the Centers for the Fight Against Cancer, *AJCC* American Joint Committee on Cancer

^a^Including fibrosarcoma, synoviosarcoma, rhabdomyosarcoma, solitary fibrous tumor

### TLR and survival

**Fig. 2:**
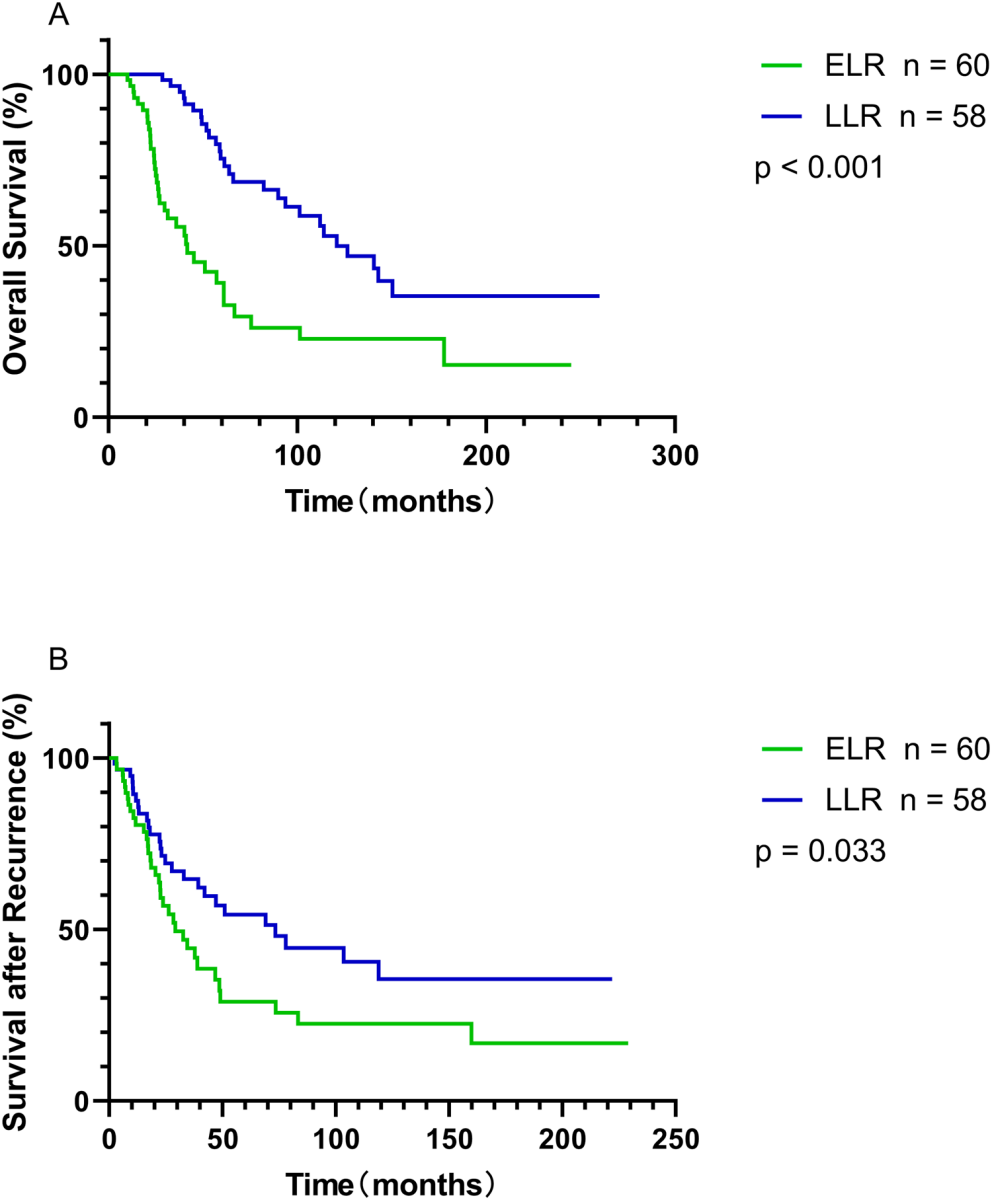
Impact of TTR on survival of patients with RPS. **A** Overall survival (*P* < 0.001) and (**B**) survival after recurrence (*P* = 0.033) curves showed that there was a worse prognosis for patients in the ELR group than those in the LLR group

At a median follow-up time of 60.5 months (IQR 33.3–142.4 months), 67 deaths (29.9%) occurred from any causes. For the entire cohort, the median OS time was 143.0 months, with 3-year OS of 84.8% and 5-year OS rate of 72.4%. The cutoff value of the TLR was defined as 20 months based on the median TLR (19.6 months). Then, the patients with local recurrence were divided into two groups, the group of ELR (TLR < 20 months, *n* = 60, 50.8%) and the group of LLR (TLR ≥ 20 months, *n* = 58, 49.2%). Subsequent analysis revealed that the ELR group presented inferior 5-year OS rates in comparison to the LLR group (35.9% vs. 73.2%, *P* = 0.004). Moreover, the median OS time and median SAR time of the patients with LLR were 120.9 months and 73.4 months, which were significantly longer than those of the patients with ELR (41.8 months and 29.2 months; *P* < 0.001, *P* = 0.033, respectively) (Fig. 2).

**Fig. 3:**
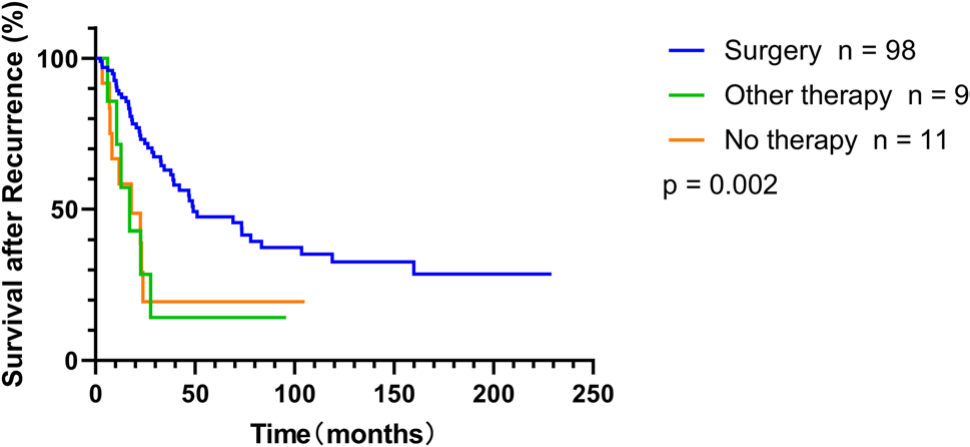
The curves showed that the patients who underwent surgery after recurrence exhibited significantly better SAR than the patients that did not receive surgery or treatment (*P* = 0.002)

In the univariate analysis of SAR and OS, it was found that TLR, tumor grade, and AJCC stage were prognostic factors. Multivariate analysis using the variables selected from univariate analysis as covariates demonstrated that TLR and tumor grade remained independent predictors for both SAR (*P* = 0.014 and *P* = 0.002, respectively) and OS (*P* < 0.001 and *P* = 0.004, respectively), but the AJCC stage was not statistically significant. Specifically, the risk of succumbing after local recurrence for patients with ELR was > 2 times higher than that for patients with LLR (HR = 2.01, 95% CI 1.15–3.50, *P* = 0.014) (Tables 3, 4). It is also worth noting that non-surgical treatment served as a negative prognostic factor for SAR (no therapy vs. surgery, HR = 2.57, 95% CI 1.16–5.70; other therapy vs. surgery, HR = 4.63, 95% CI 1.93–11.11, *P* = 0.001) (Table 3, Fig. 3).

**Table 3:** Univariate and multivariate analyses to determine independent predictors for SAR of RPS

Variables	Survival after recurrence						
	Univariate				Multivariate		
	HR	95% CI	*P* value		HR	95% CI	*P* value
Gender (male vs. female)	1.020	0.618–1.684	0.938				
Age at recurrence (≥ 60 years vs. < 60 years)	1.155	0.699–1.910	0.574				
Maximal size of tumor ((≥ 15 cm vs. < 15 cm)	1.003	0.609–1.652	0.990				
Histology subtypes			0.972				
DDLPS/MLPS vs. non-liposarcoma^a^	0.941	0.505–1.754					
WDLPS vs. non-liposarcoma^a^	1.003	0.529–1.903					
FNCLCC grade			0.001				0.002
G2 vs. G1	2.736	0.954–7.848			2.077	0.704–6.124	
G3 vs. G1	5.360	1.886–15.232			4.400	1.532–12.638	
AJCC stage			0.014				
II vs. I	6.039	1.803–20.224					
III vs. I	3.518	1.269–9.753					
Metastases after recurrence (yes vs. no)	1.381	0.625–3.050	0.425				
Therapy after recurrence			0.003				0.001
No therapy vs. surgery	2.632	1.234–5.609			2.568	1.157–5.698	
Non-surgical therapy^b^ vs. surgery	2.935	1.313–6.560			4.634	1.933–11.110	
TLR (ELR vs. LLR)	1.722	1.039–2.854	0.035		2.009	1.154–3.495	0.014

*SAR* survival after recurrence, *RPS* retroperitoneal sarcoma, *HR* hazard ratio, *95% CI* 95% confidence interval, *DDLPS* dedifferentiated liposarcoma, *MLPS* myxoid liposarcoma, *WDLPS* well-differentiated liposarcoma, *FNCLCC* French National Federation of the Centers for the Fight Against Cancer, *AJCC* American Joint Committee on Cancer, *HR* hazard ratio, *CI* confidence interval, *ELR* early local recurrence, *LLR* late local recurrence

^a^Including fibrosarcoma, synoviosarcoma, rhabdomyosarcoma, solitary fibrous tumor

^b^Including chemotherapy, radiotherapy, chemoradiotherapy, and targeted therapy

**Table 4:** Univariate and multivariate analyses to determine independent predictors for OS of RPS

Variables	Overall survival						
	Univariate				Multivariate		
	HR	95% CI	*P* value		HR	95% CI	*P* value
Gender (male vs. female)	1.072	0.663–1.733	0.777				
Age at operation (≥ 60 years vs. < 60 years)	1.741	1.049–2.890	0.032				
Maximal size of tumor (continuous)	0.984	0.956–1.012	0.253				
Maximal size of tumor (≥ 15 cm vs. < 15 cm)	0.953	0.590–1.542	0.846				
Multifocality (yes vs. no)	1.978	1.166–3.338	0.011				
Number of resected organs (≥ 1 vs. 0)	0.685	0.418–1.122	0.133				
Histology subtypes			0.987				
DDLPS/MLPS vs. non-liposarcoma^a^	1.047	0.585–1.870					
WDLPS vs. non-liposarcoma^a^	1.009	0.555–1.837					
FNCLCC grade			< 0.001				0.004
G2 vs. G1	3.507	1.356–9.068			2.917	1.125–7.564	
G3 vs. G1	7.439	2.895–19.117			4.628	1.789–11.977	
AJCC stage			0.003				
II vs. I	5.796	1.934–17.367					
III vs. I	4.613	1.841–11.555					
Postoperative therapy (yes vs. no)	1.257	0.706–2.237	0.436				
Recurrence			< 0.001				< 0.001
LLR vs. free	5.740	2.207–14.926			4.696	1.797–12.271	
ELR vs. free	15.703	6.142–40.143			12.170	4.715–31.414	

*OS* overall survival, *RPS* retroperitoneal sarcoma, *HR* hazard ratio, *95% CI* 95% confidence interval, *DDLPS* dedifferentiated liposarcoma, *MLPS* myxoid liposarcoma, *WDLPS* well-differentiated liposarcoma, *FNCLCC* French National Federation of the Centers for the Fight Against Cancer, *AJCC* American Joint Committee on Cancer, *ELR* early local recurrence, *LLR* late local recurrence

^a^Including fibrosarcoma, synoviosarcoma, rhabdomyosarcoma, solitary fibrous tumor

**Fig. 4:**
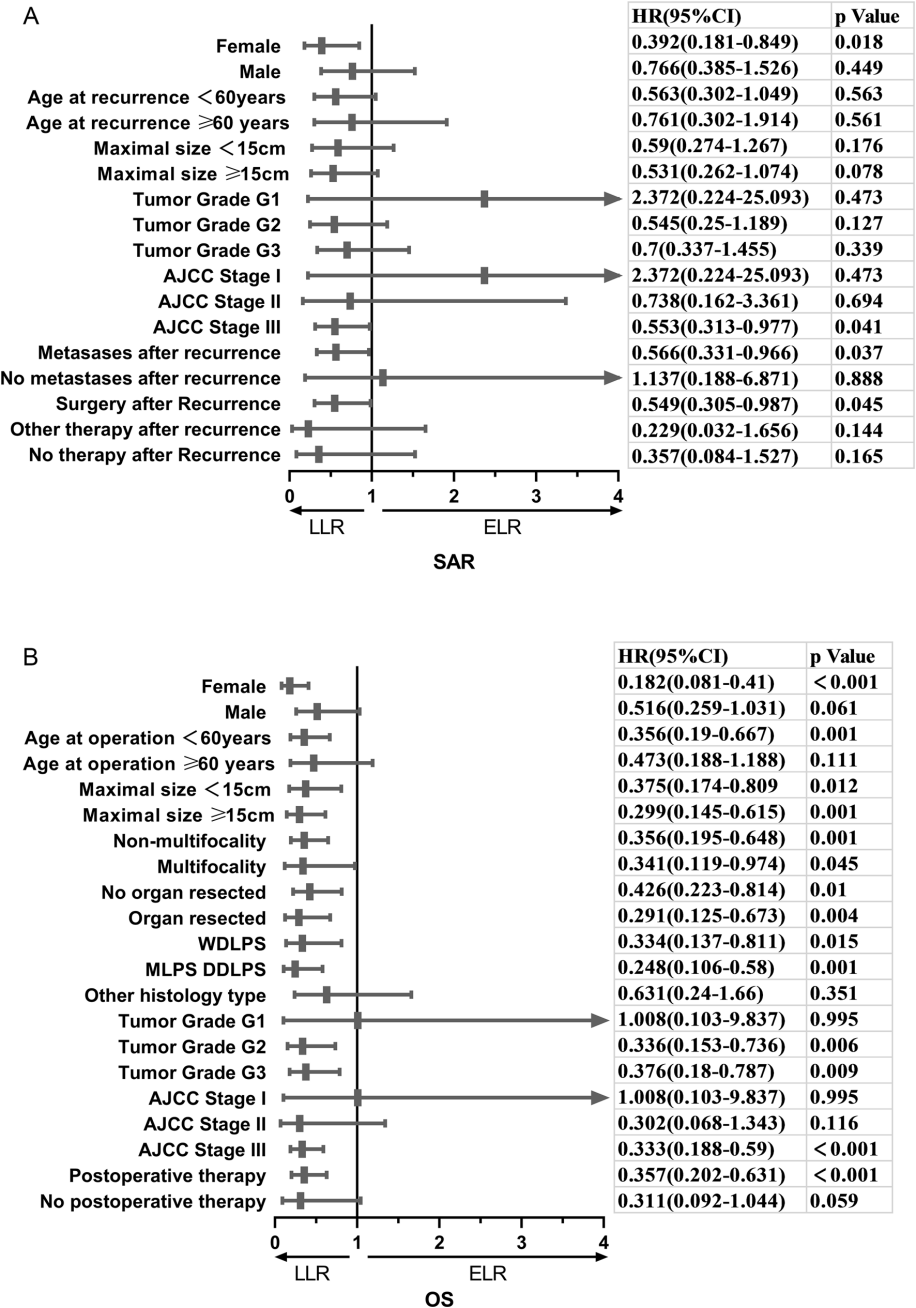
The forest plot of prognostic relevance of TTR and relevant clinicopathological characteristics using the Cox proportional hazards model. **A** The forest plot showed that ELR was an independent risk factor of SAR for patients in the subgroups of female, tumor maximal size over 15 cm, AJCC stage III, metastases after recurrence, and surgery after recurrence. **B** The forest plot showed that the ELR was an independent risk factor of OS for patients in the subgroups of female, age at operation under 60 years, any tumor maximal size, any number of tumor, any organ resection, LPS, tumor grade G2 and G3, AJCC stage III, and postoperative therapy

The prognostic relevance of TLR and the clinical pathological characteristics were compared by the Cox proportional hazards model. There were no statistically significant differences in SAR between the two groups regardless of age, tumor size, or tumor grade. It is worth noting, however, that the patients with AJCC stage III (HR = 0.55, 95% CI 0.31–0.98, *P* = 0.041) or with surgery after recurrence (HR = 0.55, 95% CI 0.31–0.99, *P* = 0.045) in the ELR group exhibited significantly worse SAR performance than those in the LLR group (Fig. 4A). As for the comparison of OS, the patients with ELR exhibited worse prognosis except for a subgroup of males, aged over 60 years, non-liposarcoma, G1, stage I/II, and without postoperative therapy after initial R0 surgery (Fig. 4B).

## Discussion

The role of TLR on survival of a variety of cancers, including breast cancer, rectal cancer, and gastric cancer [13, 19–21], has long been debated, whereas there is no research on this subject in RPS. To the best of our knowledge, this is the first study to investigate the potential impact of TLR on the outcomes of patients with STS of the retroperitoneum.

Several studies have shown that local recurrence is associated with shorter OS and progression-free survival in patients with STS of the extremity and trunk wall [9, 22–24]. In our long-term follow-up of 224 RPS patients who underwent complete surgery, local recurrence proved to be an independent prognostic factor for poor OS, which was consistent with other previously published studies [25]. In addition, local recurrence was found in more than half of the patients in our study, despite similarity to the published data [3, 6], which was obviously higher than that for other site sarcomas [26]. There are several reasons to explain this phenomenon. The symptoms of RPS are usually mild, resulting in missed best surgical opportunities at the time of discovery and diagnosis [30]. More than two-thirds of the tumors in this study were of high grade (G2–G3) or advanced TNM stage (IIIA-IIIB) at diagnosis, with tumor diameter greater than 10 cm. However, the deep anatomical position of the retroperitoneum is close to the surrounding solid organs, resulting in difficulty of surgical resection that is significantly higher than that of the extremity sarcoma, and thus, there may be invisible residual lesions after surgery [27].

In the current study, we used multivariate analysis to determine that superior LRFS was well correlated with single-focal tumors, non-liposarcoma pathological types, and G1 grade. Multifocality is a well-known factor for poor prognosis of RPS. In a retrospective study involving 393 patients with primary or recurrent RPS, it was found that patients with additional tumors exhibited worse OS, especially when the number of tumors was greater than seven [28]. Moreover, Nizri et al. noted a phenomenon whereby the number of patients with multifocal disease in recurrent RPS was much larger than that in primary RPS, which further proves that multifocal disease is a major risk factor for recurrence [29]. Compared to the report conducted by Chou et al. [30], our study is the largest retrospective study of RPS cases from a single center from Asia and is one of the few studies to demonstrate that multifocal disease is an independent prognostic factor for LRFS in RPS patients. In addition, as the most common pathological type of RPS, liposarcoma can be divided into four types according to the different morphologies, among which invasion ability and the rate recurrence of DDLPS were significantly higher than those of other histologic subtypes [25, 31, 32]. We found that the histologic subtype of non-liposarcoma conferred higher LRFS than liposarcoma, which may aid in more accurate patient counseling and selection of patients for adjuvant therapy trials.

Moreover, our results first confirmed that TLR can be an independent predictor of poor SAR and OS for RPS, and patients with LLR have longer SAR and OS time than ELR patients, especially for patients diagnosed with liposarcoma, high FNCLCC grade, and advanced TNM stage. However, the exact mechanisms by which TLR influences the clinical features and prognosis of RPS patients remain unclear. One potential reason for the negative impact of ELR on survival is that patients with ELR may be at increased risk of circulating tumor cells spreading via the hematological route or by lymphatic infiltration, with subsequent invasion of the surrounding organs.

For recurrent RPS, the selection of appropriate treatment is very important to prolong the survival time and improve the quality of life for patients. It has been reported that a second operation improves survival for RPS patients compared to biopsy or supportive treatment, even if only R2 excision is achieved without R0 or R1 excision [33]. Lenhert et al. reported that secondary complete resection of RPS patients after recurrence can achieve relatively optimistic long-term survival, which is similar to the results of this study [34]. The results of a recent multi-center retrospective study further confirmed that surgical resection is a relatively safe and effective treatment for first-time recurrent RPS [35], although radiotherapy, chemotherapy, and immunotherapy may also be appropriate treatment options. According to our results, even compared with cessation of treatment, these adjuvant supportive therapies do not improve the prognosis. However, in the recent decade, novel anti-cancer agents were introduced and had stepped up to systematic treatment. Current first-line systemic therapy for unresectable recurrent RPS is based on anthracycline regimens, while pazopanib and eribulin are recommended for subsequent line therapy [7]. We look forward to more innovative and alternative therapies that could prolong the SAR for patients.

Tumor size has been considered as a significant risk factor for OS in RPS. Nevertheless, as the present standard to evaluate the malignancy of STS, FNCLCC tumor grade was found to be an independent prognostic factor of SAR and OS in our cohort, with a far greater impact than tumor size. Unexpectedly, the AJCC stage was not also associated with local recurrence, OS, or SAR in our analysis. A recent analysis showed that the TNM staging by the AJCC in version 8 was worse than that in version 7 for predicting prognosis for RPS [36]. The decreased predictive accuracy and differential ability as described in the 8th edition of AJCC staging may be due to the overemphasis on tumor size and T staging, while ignoring histological grade and number of lesions.

There are limitations to this study, despite its important findings. First, this was a retrospective study with a wide time span for the follow-up, which may introduce existing selection bias and confounding bias in the statistical analysis. Second, the histologic pathological assessment could be controversial, as pathologists made diagnosis according to early and varied experience. We reassessed a part of the specimens, yet not all of them due to the loss of the wax blocks over long-term storage. Molecular assessment was also not performed. In addition, the clinicopathological data for some patients was incomplete. For example, we failed to describe the details of the recurrent tumor characteristics, the extent of surgery for local recurrence, and the specific chemotherapy regimens to provide reference for treatment due to the large spans of time. In addition, the small number of cases, which were restricted to the low incidence of RPS, might impact the statistical power. Although there were more patients in this study than most of the other single-center studies, our conclusions still require further verification in a larger population of LPS patients from multiple centers.

## Conclusion

Our results showed that there was a significant association between local recurrence and OS decline in adult RPS patients who underwent radical resection, and those with multifocality, liposarcoma type, or a higher tumor grade were more likely than others to undergo local recurrence. Aggressive secondary surgical treatment following local recurrence may improve long-term survival. In addition, the patients with LLR exhibited more optimal performance in both SAR and OS than the patients with ELR, and TLR was an independent prognostic factor for SAR and OS. If confirmed in a larger multi-center study, the findings of this pilot study may provide a basis for developing individualized surveillance protocols for high-risk patients that provide early diagnosis and more effective second-chance treatment in the event of relapse.

## Supplementary Information

Below is the link to the electronic supplementary material.

Supplementary file1 (DOCX 24 KB)

Supplementary file2 (TIF 51 KB)

Supplementary file3 (TIF 414 KB)
