# Supplementary material for: Impact of time to first relapse on long-term outcome in adult retroperitoneal sarcoma patients after radical resection
Source: Int J Clin Oncol. 2022 Jun 28;27(9):1487–98. doi: 10.1007/s10147-022-02205-w (PMC9393154; doi:10.1007/s10147-022-02205-w)
Supplement: Supplementary file 1 — Supplementary file1 (DOCX 24 KB) [file 10147_2022_2205_MOESM1_ESM.docx]

**Table S1** Comparison of clinicopathological characteristics between pathological types

| Variables | Histologic subtype, no. (%) | | | |
| --- | --- | --- | --- | --- |
|  | DDLPS/MLPS | WDLPS | Non-liposarcoma | *p*-value |
| Gender |  |  |  | 0.082 |
| Male | 42 (46.7) | 27 (42.2) | 43 (61.4) |  |
| Female | 48 (53.3) | 37 (57.8) | 27 (38.6) |  |
| Age at operation (years) |  |  |  | 0.108 |
| <60 | 57 (63.3) | 43 (67.2) | 55 (78.6) |  |
| ≥60 | 33 (36.7) | 21 (32.8) | 15 (21.4) |  |
| Maximal size of tumor (cm) |  |  |  | <0.001 |
| <15 | 37 (41.1) | 26 (40.6) | 54 (77.1) |  |
| ≥15 | 53 (58.9) | 38 (59.4) | 16 (22.9) |  |
| Multifocality (yes vs. no) |  |  |  | 0.203 |
| No | 73 (81.1) | 51 (79.7) | 63 (90.0) |  |
| Yes | 17 (18.9) | 13 (20.3) | 7 (10.0) |  |
| Number of resected organs |  |  |  | 0.814 |
| 0 | 47 (52.2) | 34 (53.1) | 40 (57.1) |  |
| ≥1 | 43 (47.8) | 30 (46.9) | 30 (42.9) |  |
| FNCLCC grade |  |  |  | <0.001 |
| G1 | 7 (7.8) | 40 (62.5) | 6 (8.6) |  |
| G2 | 46 (51.1) | 17 (26.6) | 38 (54.3) |  |
| G3 | 37 (41.1) | 7 (10.9) | 26 (37.1) |  |
| AJCC stage |  |  |  | <0.001 |
| I | 7 (7.8) | 38 (59.4) | 6 (8.6) |  |
| II | 5 (5.6) | 4 (6.3) | 11 (15.7) |  |
| III | 78 (86.7) | 22 (34.4) | 53 (75.7) |  |
| Postoperative therapy |  |  |  | <0.001 |
| No | 82 (91.1) | 51 (79.7) | 44 (62.9) |  |
| Yes | 8 (8.9) | 13 (20.3) | 26 (37.1) |  |
| Recurrence |  |  |  | 0.075 |
| No | 38 (42.2) | 27 (42.2) | 41 (58.6) |  |
| Yes | 52 (57.8) | 37 (57.8) | 29 (41.4) |  |
| ELR | 28 (53.8) | 19 (51.4) | 13 (44.8) | 0.737 |
| LLR | 24 (46.2) | 18 (48.6) | 16 (55.2) |  |
| Metastasis |  |  |  | <0.001 |
| No | 83 (92.2) | 62 (96.9) | 53 (75.7) |  |
| Yes | 7 (7.8) | 2 (3.1) | 17 (24.3) |  |
| Status |  |  |  | 0.675 |
| Alive | 66 (73.3) | 43 (67.2) | 48 (68.6) |  |
| Dead | 24 (26.7) | 21 (32.8) | 22 (31.4) |  |
| Median OS (IQR) | 39.64 (39.52) | 50.37 (69.17) | 52.47 (72.55) | 0.112 |
| Median SAR (IQR) | 21.25 (39.00) | 26.25 (59.27) | 28.38 (61.11) | 0.469 |
| 5-year OS | 70.7% | 71.3% | 69.8% | 0.877 |
| 3-year LRFS | 52.7% | 57.8% | 64.4% | 0.013 |

Abbreviation: DDLPS, dedifferentiated liposarcoma; MLPS, myxoid liposarcoma; WDLPS, well-differentiated liposarcoma; FNCLCC, French National Federation of the Centers for the Fight Against Cancer; AJCC, American Joint Committee on Cancer; ELR, early local recurrence; LLR, late local recurrence; SAR, survival after recurrence; IQR, interquartile range; OS, overall survival; LRFS, local recurrence-free survival

a: Including fibrosarcoma, synoviosarcoma, rhabdomyosarcoma, solitary fibrous tumor.
