# Supplementary figures and images for: Impact of time to first relapse on long-term outcome in adult retroperitoneal sarcoma patients after radical resection
Source: Int J Clin Oncol. 2022 Jun 28;27(9):1487–98. doi: 10.1007/s10147-022-02205-w (PMC9393154; doi:10.1007/s10147-022-02205-w)

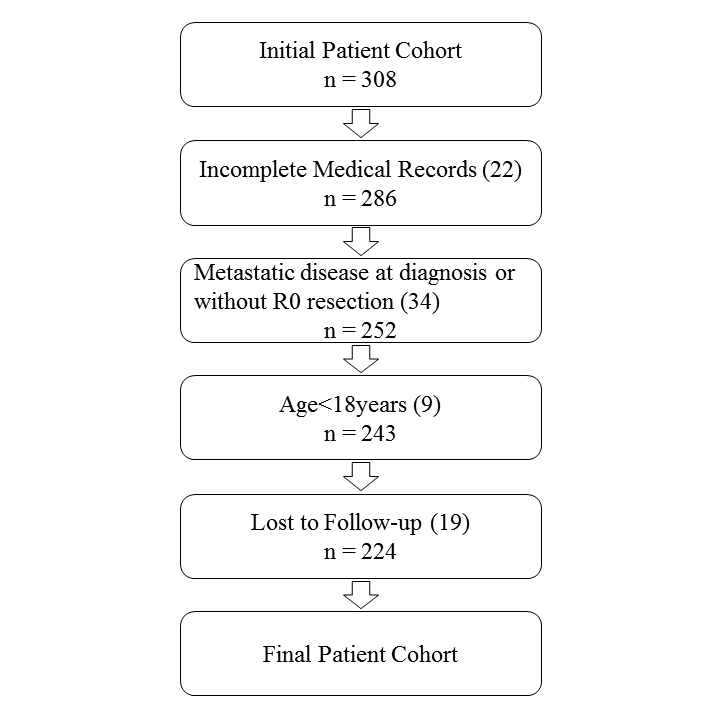

Supplement: Supplementary file 2 — Supplementary file2 (TIF 51 KB) [file 10147_2022_2205_MOESM2_ESM.tif]

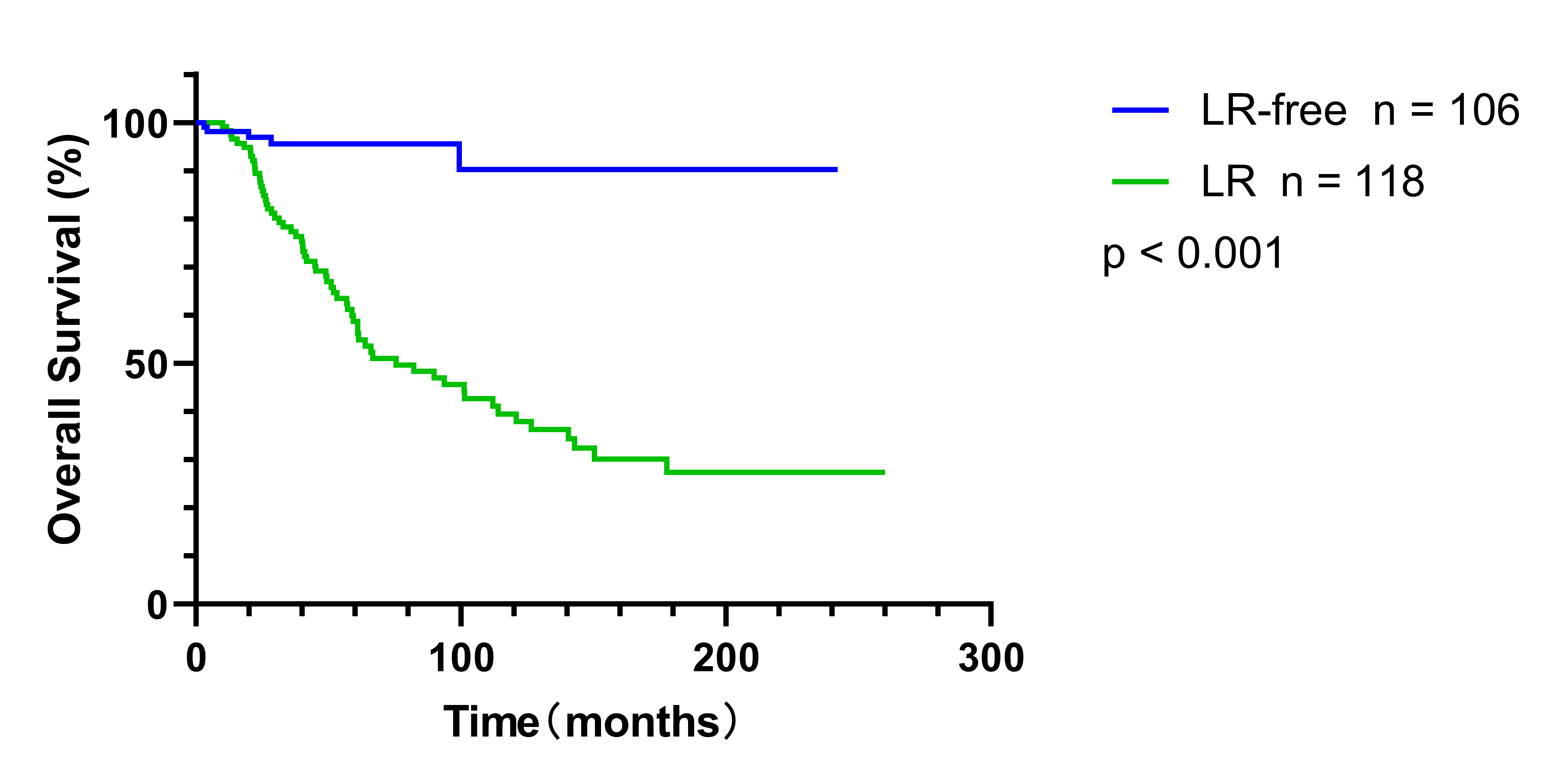

Supplement: Supplementary file 3 — Supplementary file3 (TIF 414 KB) [file 10147_2022_2205_MOESM3_ESM.tif]
